# Crystal structure and Hirshfeld surface analysis of 3,4-di­hydro-2*H*-anthra[1,2-*b*][1,4]dioxepine-8,13-dione

**DOI:** 10.1107/S2056989020003965

**Published:** 2020-03-27

**Authors:** Sofia Zazouli, Mohammed Chigr, Ahmed Jouaiti, Nathalie Kyritsakas, El Mostafa Ketatni

**Affiliations:** a Laboratory of Sustainable Development, Sultan Moulay Slimane University, Faculty of Sciences and Technologies, BP 523, 23000 Beni-Mellal, Morocco; b Laboratory of Organic and Analytical Chemistry, University Sultan Moulay Slimane, Faculty of Science and Technology, PO Box 523, Beni-Mellal, Morocco; c Molecular Tectonics Laboratory, Université de Strasbourg, CNRS, CMC UMR 7140, F-67000 Strasbourg, France

**Keywords:** crystal structure, anthra­quinone, dioxepine, Schiff base, Hirshfeld surface analysis

## Abstract

The title compound, C_17_H_12_O_4_, was synthesized from the dye alizarin. The dihedral angle between the mean plane of the anthra­quinone ring system (r.m.s. deviation = 0.039 Å) and the dioxepine ring is 16.29 (8)°. In the crystal, the mol­ecules are linked by C—H⋯O hydrogen bonds, forming sheets lying parallel to the *ab* plane. The sheets are connected through π–π and C=O⋯π inter­actions to generate a three-dimensional supra­molecular network. Hirshfeld surface analysis was used to investigate inter­molecular inter­actions in the solid-state: the most important contributions are from H⋯H (43.0%), H⋯O/O⋯H (27%), H⋯C/C⋯H (13.8%) and C⋯C (12.4%) contacts.

## Abstract 2

The dihedral angle between the mean plane of the anthra­quinone ring system and the dioxepine ring in the title compound is 16.29 (8)°. The packing is consolidated by C—H⋯O, π–π and C=O⋯π inter­actions.

## Chemical context   

Anthra­quinone derivatives, which are extracted from the seeds of the Rubiaceae family of shrubs, include alizarin (1,2-di­hydroxy­anthra­quinone; C_14_H_8_O_4_) and other polycyclic aromatic hydro­carbons. The colour of anthra­quinone-based compounds can be modified by the type and position of the substituents attached to the anthra­quinone nucleus (Nakagawa *et al.* 2017 ▸; Cheuk *et al.*, 2015 ▸; Tonin *et al.*, 2017 ▸). Besides their application as pigments or dyes in textile, photographic, cosmetic and other industries (Wang *et al.*, 2011 ▸), anthra­quinone derivatives have been used for centuries for medical applications, for example, as laxatives (Oshio *et al.*, 1985 ▸), anti­oxidants (Yen *et al.*, 2000 ▸), anti­microbial (Xiang *et al.*, 2008 ▸; Yadav *et al.*, 2010 ▸) and anitiviral (Alves *et al.*, 2004 ▸) agents. Their redox properties and cytotoxicity have been investigated recently (Okumura *et al.*, 2019 ▸). Anthra­quinone derivatives exhibit various applications in supra­molecular and electro-analytical chemistry (Czupryniak *et al.*, 2012 ▸).
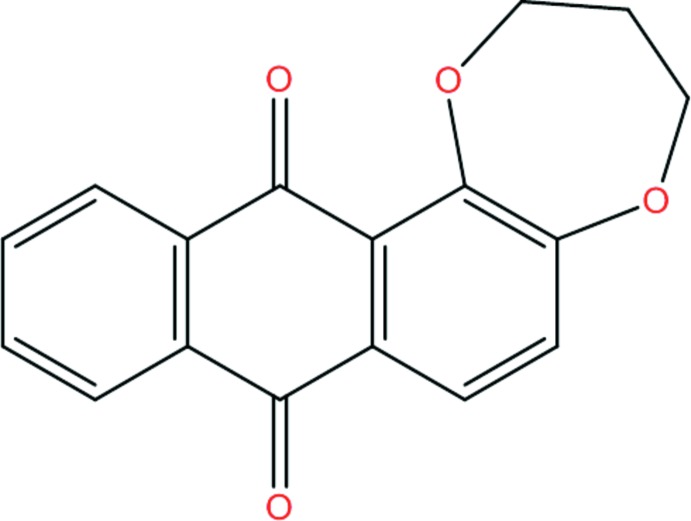


As part of our studies in this area, the synthesis and structure of the title compound, (I), are described along with a detailed analysis of its supra­molecular associations through an analysis of the Hirshfeld surfaces.

## Structural commentary   

**Figure 1:**
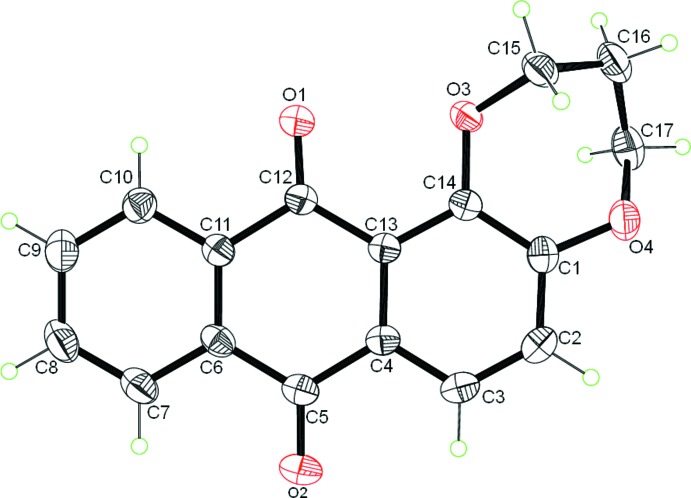
The mol­ecular structure of (I) with displacement ellipsoids drawn at the 50% probability level.

Compound (I) crystallizes in space group *P*2_1_/*n* with one mol­ecule in the asymmetric unit: it consists of three fused six-membered rings and one seven-membered ring as shown in Fig. 1 ▸. The fused-ring system is close to planar with an r.m.s. deviation for all non-hydrogen atoms of 0.039 Å (the dihedral angle between the aromatic rings of the anthra­quinone unit and the central ring range from 1.5 to 1.9°). The dioxepine ring is inclined to the mean plane of the anthra­quinone ring system by 16.29 (8)°.

A puckering analysis of the seven-membered ring yielded the parameters *q2* = 0.896 (2) Å, φ_2_ = 113.50 (12)°, *q*_3_ = 0.358 (2) Å, and φ_3_ = 217.8 (3)°. These metrics indicate that the ring adopts a screw boat conformation. The C—O and C=O bond lengths lie within the ranges 1.355 (2)–1.457 (2) Å and 1.216 (2)–1.226 (2) Å, respectively, confirming their single and double-bond character.

## Supra­molecular features   

**Figure 2:**
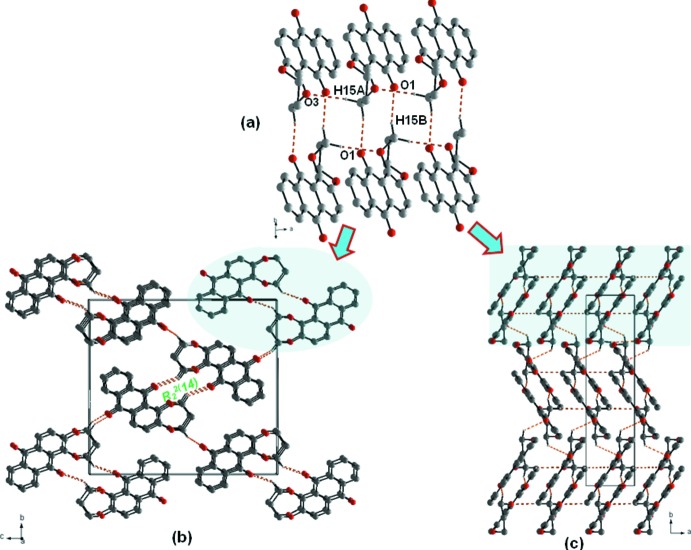
(*a*) Inversion dimers with 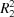
(14) ring motifs; (*b*) and (*c*) packing diagrams of the title compound, viewed along the *a* and *b* axes, respectively. Dotted lines indicate C—H⋯O hydrogen bonds.

**Figure 3:**
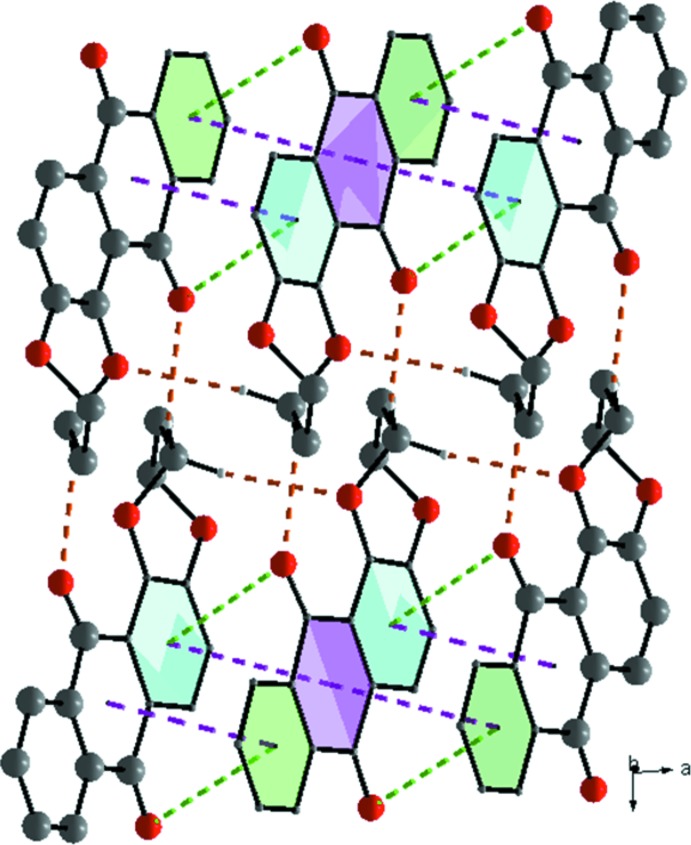
Partial crystal packing for (I) showing the C—H⋯O hydrogen bonds and the offset π–π (purple) and C=O⋯π (green) inter­actions between inversion-related mol­ecules.

In the extended structure of (I), C15—H15*B*⋯O1 hydrogen bonds form inversion dimers with an 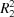
(14) ring motif. Adjacent dimers are linked by C15—H15*A*⋯O3 contacts, thereby generating corrugated chains of mol­ecules (Fig. 2 ▸ *a*). A C17—H17*B*⋯O2 hydrogen bond links the chains together (Table 1 ▸; Fig. 2 ▸ *b* and 2*c*), forming sheets propagating in the *ab* plane. These sheets are supported by extensive π–π contacts between adjacent rings, with centroid-to-centroid distances *Cg*1⋯*Cg*2 = 3.599 (2) and *Cg*2⋯*Cg*3 = 3.683 (2) Å [*Cg*1, *Cg*2 and *Cg*3 are the centroids of the rings C1–C4/C13–C14, C4–C6/C11–C13 and C6–C11, respectively] and weak C12=O1⋯π [oxygen–centroid distance = 3.734 (2) Å] inter­actions (Fig. 3 ▸), linking the slabs to form a three-dimensional supra­molecular network.

## Database survey   

A search in the Cambridge Structural Database (CSD, Version 5.40, updated to February 2020; Groom *et al.*, 2016 ▸) revealed 55 alizarin-ring motifs incorporated in more complex mol­ecules or bearing functional groups. These include several compounds with a different substituent in place of the dioxepine in the title compound, *viz*. 1-hy­droxy-2-meth­oxy-6-methyl (BOTXUE; Ismail *et al.*, 2009 ▸), 1,2-dimeth­oxy (refcode: KIBHUZ; Kar *et al.*, 2007 ▸) and 3-hy­droxy-1,2-dimeth­oxy (BOVVEO; Xu *et al.*, 2009 ▸). In these compounds, the anthra­quinone ring system are almost planar, the dihedral angle between the benzene rings for BOTXUE, KIBHUZ and BOVVEO being 3.49, 2.83 and 1.12°, respectively. The meth­oxy groups in position 1 (C14) in KIBHUZ and BOVVEO are almost perpendicular to the anthra­quinone ring plane. The other compound belongs to the same class of alizarins with different substituents.

## Hirshfeld surface analysis   

**Figure 4:**
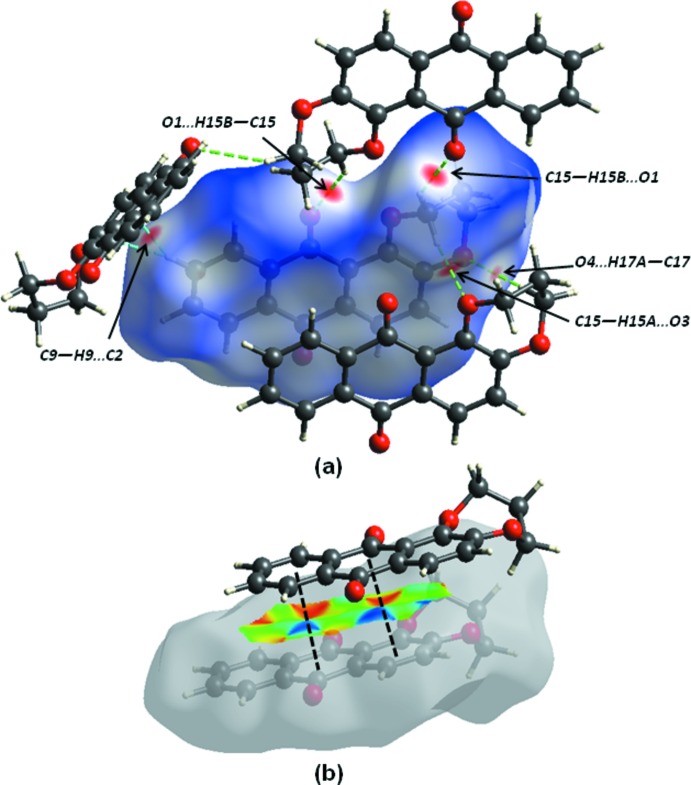
Views of the Hirshfeld surface for (I) mapped over (*a*) *d*_norm_ showing the C—H⋯O contacts as green dashed lines and short C⋯H/H⋯C contacts as cyan dashed lines; and (*b*) shape-index highlighting the π–π stacking (black lines).

The nature of the inter­molecular inter­actions in (I) have been examined with *CrystalExplorer17.5* (Turner *et al.*, 2017 ▸), using Hirshfeld surface analysis (Spackman & Jayatilaka, 2009 ▸) mapped over *d*_norm_, with a fixed colour scale of −0.1779 to 1.3612 a.u (see Fig. S1*a* in the supporting information) and two-dimensional fingerprint plots (McKinnon *et al.*, 2007 ▸). The intense red spots on the surface are due to the C—H⋯O hydrogen bonds (Fig. 4 ▸). Fig. S2 (supporting information) shows the mol­ecular electrostatic potential surface generated using *TONTO* with a STO-3G basis set in the range −0.050 to 0.050 a.u. within the Hartree–Fock level of theory. Mol­ecular sites evidenced in red correspond to positive potential energy and in blue to negative potential energy (Spackman *et al.*, 2008 ▸).

**Figure 5:**
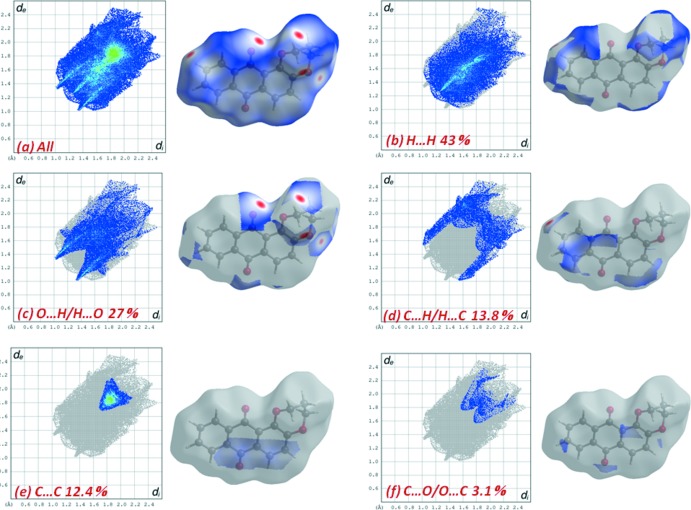
The full two-dimensional fingerprint plots for (I) showing (*a*) all inter­actions, and delineated into (*b*) H⋯H, (*c*) H⋯O/O⋯H, (*d*) H⋯C/C⋯H, (*e*) C⋯C and (*f*) O⋯C/C⋯O inter­actions.

As illustrated in Fig. 5 ▸, the overall fingerprint plot for (I) and those delineated into H⋯H, H⋯O/O⋯H, C⋯H/H⋯C and C⋯C show characteristic pseudo-symmetric wings in the *d*_e_ and *d*_i_ diagonal axes. The most important inter­action is H⋯H, contributing 43% to the overall crystal packing, which is reflected in Fig. 5 ▸ *b* as widely scattered points of high density due to the large hydrogen content of the mol­ecule, with small split tips at *d*_e_ ≃ *d*_i_ ≃ 1.2 Å. The contribution from the O⋯H/H⋯O contacts (27%) [note that the O⋯H interactions make a larger contribution (14.6%) than the H⋯O interactions (12.4%)], corresponding to C—H⋯O inter­actions, is represented by a pair of sharp spikes characteristic of a strong hydrogen-bond inter­action, *d*_e_ + *d*_i_ ≃ 2.35 Å (Fig. 5 ▸ *c*). The significant contribution from C⋯H/H⋯C contacts (13.8%) to the Hirshfeld surface of (I) reflect the short C⋯H/H⋯C contacts, and the distribution of points has characteristic wings, Fig. 5 ▸ *d*, with *d*_e_ + *d*_i_ ≃2.55 Å. The distribution of points in the *d*_e_ = d_i_ ≃ 1.75 Å range in the fingerprint plot delineated into C⋯C contacts indicates the existence of weak π–π stacking inter­actions between the central anthracene ring and the C6–C11 and C1–C4/C13–C14 rings (Fig. 4 ▸ *b* and 5*e*). Aromatic π–π inter­actions are indicated by adjacent red and blue triangles in the shape-index map (Fig. S1*b*)and also by the flat region around these rings in the Hirshfeld surfaces mapped over curvedness in Fig. S1*c*.

The contribution of 3.2% from C⋯O/O⋯C contacts is due to the presence of short inter­atomic C=O⋯π contacts, and is apparent as the pair of parabolic tips at *d*_e_ + *d*_i_ ≃ 3.2 Å in Fig. 5 ▸ *f*.

## Synthesis and crystallization   

**Figure 6:**
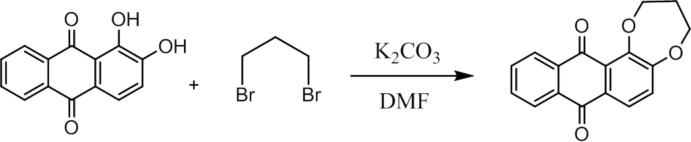
Synthesis pathway leading to the formation of the title compound.

Under argon, alizarin (0.50 g, 2.0 mmol) was treated with 1,3-di­bromo-propane (0.42 g, 2.0 mmol) in di­methyl­formamide (30 ml) in the presence of anhydrous potassium carbonate (1.0 g, 7.2 mmol) with stirring and heated to 393 K for 24 h. The reaction mixture was evaporated to dryness under vacuum and the resulting crude product was acidified with 12 *N* hydro­chloric acid, extracted with chloro­form (3 × 30 ml) and then chromatographed on a silica gel column with di­chloro­methane/petroleum ether (1/1) as eluent, which yielded 200 mg (35%) of 1,2-propyl­ene­dioxy­anthra­quinone as a yellow compound (Fig. 6 ▸). Colourless needles were obtained by slow evaporation of a di­chloro­methane/petroleum ether (1:1) solution.

^1^H NMR (CDCl_3_, 500 MHz): δ (ppm): 8.21 (*m*, 2H), 7.95 (*d*, *J* = 8.5 Hz, 1H), 7.72 (*m*, 2H), 7.26 (*d*, *J* = 8.5 Hz, 1H), 4.48 (*t*, *J* = 6 Hz, 2H), 4.43 (*t*, *J* = 6 Hz, 2H), 2.34 (*qt*, *J* = 6 Hz, 2H); ^13^C NMR (CDCl_3_, 126 MHz): δ (ppm): 182.9, 182.5, 157.3, 151.3, 135.2, 133.9, 133.4, 132.6, 129.6, 127.1, 126.5, 126.0, 125.9, 123.3, 70.5, 70.2, 30.0. Analysis calculated for C_17_H_12_O_4_: C, 72.85%; H, 4.32%; found: C, 72.82%; H, 4.29%.

## Refinement   

Crystal data, data collection and structure refinement details are summarized in Table 2 ▸. H atoms were placed in calculated positions and refined in the riding model: C—H = 0.95–0.99 Å with *U*_iso_(H) = 1.2*U*_eq_(C). The reflection (011), affected by the beam-stop, was removed during refinement.

## Supplementary Material

Crystal structure: contains datablock(s) I, global. DOI: 10.1107/S2056989020003965/hb7899sup1.cif

Structure factors: contains datablock(s) I. DOI: 10.1107/S2056989020003965/hb7899Isup2.hkl

Supporting information file. DOI: 10.1107/S2056989020003965/hb7899Isup3.cml

Supplementary figures: Hirshfeld surface analysis. DOI: 10.1107/S2056989020003965/hb7899sup4.docx

CCDC reference: 1991271

Additional supporting information:  crystallographic information; 3D view; checkCIF report

## Figures and Tables

**Table 1:** Hydrogen-bond geometry (Å, °)

*D*—H⋯*A*	*D*—H	H⋯*A*	*D*⋯*A*	*D*—H⋯*A*
C15—H15*B*⋯O1^i^	0.99	2.43	3.248 (2)	139
C15—H15*A*⋯O3^ii^	0.99	2.48	3.461 (3)	171
C17—H17*A*⋯O4^iii^	0.99	2.59	3.580 (3)	174

Symmetry codes: (i) 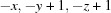; (ii) 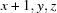; (iii) 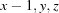.

**Table 2:** Experimental details

Crystal data	
Chemical formula	C_17_H_12_O_4_
*M*_r_	280.27
Crystal system, space group	Monoclinic, *P*2_1_/*n*
Temperature (K)	173
*a*, *b*, *c* (Å)	4.2951 (2), 16.7714 (9), 18.0537 (11)
β (°)	95.941 (2)
*V* (Å^3^)	1293.51 (12)
*Z*	4
Radiation type	Mo *K*α
μ (mm^−1^)	0.10
Crystal size (mm)	0.12 × 0.10 × 0.10

Data collection	
Diffractometer	Bruker APEXII CCD
Absorption correction	Multi-scan (*SADABS*; Bruker, 2012 ▸)
*T*_min_, *T*_max_	0.988, 0.990
No. of measured, independent and observed [*I* > 2σ(*I*)] reflections	19692, 3436, 2309
*R*_int_	0.044
(sin θ/λ)_max_ (Å^−1^)	0.697

Refinement	
*R*[*F* ^2^ > 2σ(*F* ^2^)], *wR*(*F* ^2^), *S*	0.055, 0.149, 1.03
No. of reflections	3436
No. of parameters	190
H-atom treatment	H-atom parameters constrained
Δρ_max_, Δρ_min_ (e Å^−3^)	0.41, −0.32

Computer programs: *APEX2* and *SAINT* (Bruker, 2012 ▸), *SHELXT2014/5* (Sheldrick, 2015*a* ▸), *SHELXL2018/3* (Sheldrick, 2015*b* ▸), *ORTEP-3 for Windows* (Farrugia, 2012 ▸), *DIAMOND* (Brandenburg *et al.*, 2012 ▸), *PLATON* (Spek, 2020 ▸) and *publCIF* (Westrip, 2010 ▸).

## supplementary crystallographic information

### Crystal data

**Table:**

C_17_H_12_O_4_	*F*(000) = 584
*M**_r_* = 280.27	*D*_x_ = 1.439 Mg m^−^^3^
Monoclinic, *P*2_1_/*n*	Mo *K*α radiation, λ = 0.71073 Å
*a* = 4.2951 (2) Å	Cell parameters from 3436 reflections
*b* = 16.7714 (9) Å	θ = 2.4–29.7°
*c* = 18.0537 (11) Å	µ = 0.10 mm^−^^1^
β = 95.941 (2)°	*T* = 173 K
*V* = 1293.51 (12) Å^3^	Prism, colorless
*Z* = 4	0.12 × 0.10 × 0.10 mm

### Data collection

**Table:**

Bruker APEXII CCD diffractometer	2309 reflections with *I* > 2σ(*I*)
φ and ω scans	*R*_int_ = 0.044
Absorption correction: multi-scan (SADABS; Bruker, 2012)	θ_max_ = 29.7°, θ_min_ = 2.4°
*T*_min_ = 0.988, *T*_max_ = 0.990	*h* = −5→4
19692 measured reflections	*k* = −23→23
3436 independent reflections	*l* = −24→25

### Refinement

**Table:**

Refinement on *F*^2^	Primary atom site location: dual
Least-squares matrix: full	Hydrogen site location: inferred from neighbouring sites
*R*[*F*^2^ > 2σ(*F*^2^)] = 0.055	H-atom parameters constrained
*wR*(*F*^2^) = 0.149	*w* = 1/[σ^2^(*F*_o_^2^) + (0.0548*P*)^2^ + 0.8849*P*] where *P* = (*F*_o_^2^ + 2*F*_c_^2^)/3
*S* = 1.02	(Δ/σ)_max_ < 0.001
3436 reflections	Δρ_max_ = 0.41 e Å^−^^3^
190 parameters	Δρ_min_ = −0.32 e Å^−^^3^
0 restraints	

### Special details

**Table:**

Geometry. All esds (except the esd in the dihedral angle between two l.s. planes) are estimated using the full covariance matrix. The cell esds are taken into account individually in the estimation of esds in distances, angles and torsion angles; correlations between esds in cell parameters are only used when they are defined by crystal symmetry. An approximate (isotropic) treatment of cell esds is used for estimating esds involving l.s. planes.

### Fractional atomic coordinates and isotropic or equivalent isotropic displacement parameters (Å^2^)

**Table:**

	*x*	*y*	*z*	*U*_iso_*/*U*_eq_	
O1	−0.2635 (5)	0.49906 (10)	0.64736 (8)	0.0546 (5)	
O2	0.1717 (4)	0.34277 (9)	0.89646 (7)	0.0449 (4)	
O3	0.0471 (3)	0.40325 (8)	0.56747 (6)	0.0293 (3)	
O4	0.4204 (4)	0.25897 (8)	0.56830 (7)	0.0356 (3)	
C1	0.3367 (5)	0.29496 (11)	0.63141 (10)	0.0274 (4)	
C2	0.4523 (5)	0.26018 (11)	0.69817 (11)	0.0321 (4)	
H2	0.590316	0.215932	0.697909	0.038*	
C3	0.3704 (5)	0.28874 (11)	0.76486 (10)	0.0302 (4)	
H3	0.451085	0.264092	0.810209	0.036*	
C4	0.1701 (4)	0.35347 (10)	0.76600 (9)	0.0244 (4)	
C5	0.0822 (5)	0.37964 (11)	0.83960 (10)	0.0285 (4)	
C6	−0.1143 (5)	0.45162 (10)	0.84226 (9)	0.0269 (4)	
C7	−0.1904 (5)	0.47948 (12)	0.91105 (10)	0.0351 (5)	
H7	−0.121046	0.451264	0.955331	0.042*	
C8	−0.3662 (6)	0.54788 (13)	0.91472 (11)	0.0407 (5)	
H8	−0.413992	0.567394	0.961621	0.049*	
C9	−0.4731 (5)	0.58819 (12)	0.85004 (11)	0.0377 (5)	
H9	−0.595642	0.635045	0.852781	0.045*	
C10	−0.4023 (5)	0.56056 (11)	0.78134 (11)	0.0308 (4)	
H10	−0.478159	0.588130	0.737151	0.037*	
C11	−0.2198 (4)	0.49227 (10)	0.77718 (9)	0.0248 (4)	
C12	−0.1467 (5)	0.46397 (10)	0.70231 (9)	0.0271 (4)	
C13	0.0595 (4)	0.39292 (10)	0.69919 (9)	0.0227 (4)	
C14	0.1495 (4)	0.36425 (10)	0.63107 (9)	0.0238 (4)	
C15	0.2543 (5)	0.40456 (12)	0.50847 (10)	0.0331 (4)	
H15A	0.474823	0.405280	0.530766	0.040*	
H15B	0.216063	0.453607	0.478396	0.040*	
C16	0.1997 (6)	0.33217 (13)	0.45871 (11)	0.0389 (5)	
H16A	0.001584	0.338786	0.426047	0.047*	
H16B	0.372274	0.327239	0.426649	0.047*	
C17	0.1829 (6)	0.25735 (13)	0.50493 (11)	0.0384 (5)	
H17A	−0.026930	0.253140	0.522692	0.046*	
H17B	0.214839	0.210027	0.473818	0.046*	

### Atomic displacement parameters (Å^2^)

**Table:**

	*U*^11^	*U*^22^	*U*^33^	*U*^12^	*U*^13^	*U*^23^
O1	0.0835 (14)	0.0550 (10)	0.0254 (7)	0.0370 (9)	0.0062 (8)	0.0053 (6)
O2	0.0590 (12)	0.0499 (9)	0.0257 (7)	0.0106 (8)	0.0036 (7)	0.0110 (6)
O3	0.0301 (8)	0.0378 (7)	0.0201 (6)	0.0062 (6)	0.0029 (5)	0.0009 (5)
O4	0.0345 (9)	0.0386 (8)	0.0346 (7)	0.0062 (6)	0.0078 (6)	−0.0075 (6)
C1	0.0248 (10)	0.0272 (9)	0.0307 (9)	−0.0022 (7)	0.0053 (7)	−0.0040 (7)
C2	0.0296 (11)	0.0267 (9)	0.0394 (10)	0.0036 (7)	0.0010 (8)	0.0015 (7)
C3	0.0297 (11)	0.0283 (9)	0.0313 (9)	0.0005 (7)	−0.0030 (8)	0.0054 (7)
C4	0.0245 (10)	0.0230 (8)	0.0252 (8)	−0.0033 (7)	0.0002 (7)	0.0022 (6)
C5	0.0314 (11)	0.0298 (9)	0.0237 (8)	−0.0042 (7)	0.0002 (7)	0.0022 (7)
C6	0.0309 (11)	0.0278 (8)	0.0220 (8)	−0.0070 (7)	0.0025 (7)	−0.0014 (6)
C7	0.0445 (14)	0.0376 (10)	0.0238 (9)	−0.0053 (9)	0.0064 (8)	−0.0010 (7)
C8	0.0538 (16)	0.0407 (11)	0.0295 (10)	−0.0035 (10)	0.0143 (9)	−0.0078 (8)
C9	0.0429 (14)	0.0317 (10)	0.0402 (11)	0.0007 (9)	0.0126 (9)	−0.0054 (8)
C10	0.0342 (12)	0.0264 (9)	0.0322 (9)	−0.0008 (7)	0.0054 (8)	−0.0002 (7)
C11	0.0274 (10)	0.0233 (8)	0.0239 (8)	−0.0051 (7)	0.0027 (7)	−0.0011 (6)
C12	0.0304 (11)	0.0274 (9)	0.0234 (8)	0.0015 (7)	0.0015 (7)	0.0006 (6)
C13	0.0233 (10)	0.0213 (8)	0.0231 (8)	−0.0039 (6)	0.0010 (6)	0.0006 (6)
C14	0.0218 (10)	0.0255 (8)	0.0236 (8)	−0.0033 (7)	0.0005 (7)	−0.0003 (6)
C15	0.0353 (12)	0.0407 (11)	0.0246 (9)	0.0002 (8)	0.0085 (8)	0.0014 (7)
C16	0.0396 (14)	0.0525 (12)	0.0256 (9)	0.0027 (10)	0.0080 (8)	−0.0079 (8)
C17	0.0376 (13)	0.0428 (11)	0.0357 (10)	−0.0027 (9)	0.0078 (9)	−0.0145 (8)

### Geometric parameters (Å, º)

**Table:**

O1—C12	1.216 (2)	C7—H7	0.9500
O2—C5	1.226 (2)	C8—C9	1.386 (3)
O3—C14	1.355 (2)	C8—H8	0.9500
O3—C15	1.457 (2)	C9—C10	1.387 (3)
O4—C1	1.370 (2)	C9—H9	0.9500
O4—C17	1.452 (3)	C10—C11	1.394 (3)
C1—C2	1.384 (3)	C10—H10	0.9500
C1—C14	1.413 (3)	C11—C12	1.496 (2)
C2—C3	1.375 (3)	C12—C13	1.489 (2)
C2—H2	0.9500	C13—C14	1.411 (2)
C3—C4	1.387 (3)	C15—C16	1.514 (3)
C3—H3	0.9500	C15—H15A	0.9900
C4—C13	1.414 (2)	C15—H15B	0.9900
C4—C5	1.485 (2)	C16—C17	1.513 (3)
C5—C6	1.477 (3)	C16—H16A	0.9900
C6—C11	1.393 (2)	C16—H16B	0.9900
C6—C7	1.397 (2)	C17—H17A	0.9900
C7—C8	1.379 (3)	C17—H17B	0.9900
			
C14—O3—C15	117.23 (15)	C11—C10—H10	120.0
C1—O4—C17	116.12 (16)	C6—C11—C10	119.49 (16)
O4—C1—C2	115.92 (17)	C6—C11—C12	121.76 (16)
O4—C1—C14	123.85 (16)	C10—C11—C12	118.75 (15)
C2—C1—C14	120.22 (16)	O1—C12—C13	123.57 (16)
C3—C2—C1	120.95 (18)	O1—C12—C11	118.43 (17)
C3—C2—H2	119.5	C13—C12—C11	117.99 (14)
C1—C2—H2	119.5	C14—C13—C4	119.06 (16)
C2—C3—C4	120.08 (17)	C14—C13—C12	121.55 (15)
C2—C3—H3	120.0	C4—C13—C12	119.39 (15)
C4—C3—H3	120.0	O3—C14—C13	118.68 (15)
C3—C4—C13	120.52 (16)	O3—C14—C1	122.38 (15)
C3—C4—C5	117.40 (15)	C13—C14—C1	118.92 (15)
C13—C4—C5	122.07 (16)	O3—C15—C16	110.66 (16)
O2—C5—C6	121.06 (17)	O3—C15—H15A	109.5
O2—C5—C4	120.92 (18)	C16—C15—H15A	109.5
C6—C5—C4	118.02 (15)	O3—C15—H15B	109.5
C11—C6—C7	120.04 (18)	C16—C15—H15B	109.5
C11—C6—C5	120.65 (16)	H15A—C15—H15B	108.1
C7—C6—C5	119.31 (16)	C17—C16—C15	110.55 (16)
C8—C7—C6	120.04 (18)	C17—C16—H16A	109.5
C8—C7—H7	120.0	C15—C16—H16A	109.5
C6—C7—H7	120.0	C17—C16—H16B	109.5
C7—C8—C9	120.06 (18)	C15—C16—H16B	109.5
C7—C8—H8	120.0	H16A—C16—H16B	108.1
C9—C8—H8	120.0	O4—C17—C16	110.46 (18)
C8—C9—C10	120.42 (19)	O4—C17—H17A	109.6
C8—C9—H9	119.8	C16—C17—H17A	109.6
C10—C9—H9	119.8	O4—C17—H17B	109.6
C9—C10—C11	119.94 (18)	C16—C17—H17B	109.6
C9—C10—H10	120.0	H17A—C17—H17B	108.1

### Hydrogen-bond geometry (Å, º)

**Table:**

*D*—H···*A*	*D*—H	H···*A*	*D*···*A*	*D*—H···*A*
C15—H15*B*···O1^i^	0.99	2.43	3.248 (2)	139
C15—H15*A*···O3^ii^	0.99	2.48	3.461 (3)	171
C17—H17*A*···O4^iii^	0.99	2.59	3.580 (3)	174

Symmetry codes: (i) −*x*, −*y*+1, −*z*+1; (ii) *x*+1, *y*, *z*; (iii) *x*−1, *y*, *z*.
