# Supplementary material for: Crystal structure and Hirshfeld surface analysis of 3,4-di­hydro-2H-anthra[1,2-b][1,4]dioxepine-8,13-dione
Source: Acta Crystallogr E Crystallogr Commun. 2020 Mar 27;76(Pt 4):576–80. doi: 10.1107/S2056989020003965 (PMC7133044; doi:10.1107/S2056989020003965)
Supplement: Supplementary file 4 [file e-76-00576-sup4.docx]

**Crystal structure and Hirshfeld analysis surface of 3,4-dihydro-2*H*-anthra[1,2-*b*][1,4]dioxepine-8,13-dione**

**Sofia Zazouli*^a^, Mohammed Chigr^b^, Ahmed Jouaiti^a^, Nathalie Kyritsakas^c^ and El Mostafa Ketatni^b^**

### ^a^Laboratory of Sustainable Development, Sultan Moulay Slimane University, Faculty, of Sciences and Technologies, B.P.523, 23000 BeniMellal, Morocco, ^b^Laboratory of Organic and Analytical Chemistry, University Sultan Moulay Slimane, Faculty of Science and Technology, PO Box, 523, Beni-Mellal, and ^c^Molecular Tectonics Laboratory, Université de Strasbourg, CNRS, CMC UMR 7140, F-67000 Strasbourg, France

### Correspondence e-mail: szazouli88@gmail.com

**Figure S1:**

Views of Hirshfeld surfaces of the title compound plotted over (a) *d*_norm_, (b) shape-index map and (c) curvedness map.

**Figure S2**:

View of the three-dimensional Hirshfeld surface of the title compound plotted over electrostatic potential energy in the range -0.0500 to 0.0500 a.u. using the STO-3 G basis set at the Hartree–Fock level of theory.
